# Hepatic DNA methylation and expression profiles under prenatal restricted diet in three generations of female rat fetuses

**DOI:** 10.1371/journal.pone.0215471

**Published:** 2019-04-16

**Authors:** Joanna Nowacka-Woszuk, Adrian Grzemski, Magdalena Sliwinska, Agata Chmurzynska

**Affiliations:** 1 Department of Genetics and Animal Breeding, Poznan University of Life Sciences, Wolynska Poznan, Poland; 2 Institute of Human Nutrition and Dietetics, Poznan University of Life Sciences, Wojska Polskiego, Poznan, Poland; INIA, SPAIN

## Abstract

The nutritional factors acting during early life can affect the development of the organism. It has been hypothesized that such programmed traits can be inherited by later generations. In this work, we present for the first time the effect of food deprivation in pregnant dams and its consequences for the transcription and DNA methylation profiles in the offspring of the next three generations. We used a 50% reduction in dietary intake during pregnancy in the rat and determined whether this altered the hepatic DNA methylation and transcription levels in female fetuses over three generations. Targeted next-generation sequencing (tNGS) was used in the first generation for 1748 genes associated with six selected biological processes. The selected cytosines were then studied by pyrosequencing in F1–F3. The transcript level of the selected genes was determined by the real-time PCR. The tNGS approach indicated 394 cytosines, in close proximity to 374 genes, with a statistically significant difference in methylation levels between the control and restricted groups. A gene clustering analysis revealed 23 molecular pathways to which the studied genes were assigned. Only seven cytosines were differently methylated to more than 10%, and so these sites were studied next using pyrosequencing. The observation from NGS was confirmed for only one cytosine located near the *St6galnac5* gene, though this was with the opposite effect. A difference was also observed for the *Usp30* gene, though in proximity to the cytosine selected from NGS. In F3, the differences were observed for the *Oxct2b* gene. We also found differences in methylation levels between generations for the *Grb10* and *St6galnac5* genes, but independently of the diet used. The transcript levels of selected genes (*Usp30*, *Grb10*, *Pld1*, *St6galnac5*, *Oxct2b*, *Khk*, and *Acsl4*) were not altered in F1, while changes were detected for *Pld1* and *Oxct2b* in F2 and F3, respectively. Prenatal food deprivation did not induce broad changes in hepatic DNA methylation of the genes involved in lipid or carbohydrate metabolism, and did not result in alterations in their transcription. Thus, the hypothesis that transgenerational inheritance is induced by dietary restriction was not confirmed.

## Introduction

The use of prenatal restricted diets in rodent models can be considered to be a counterpart of the Dutch Hunger Winter syndrome observed in humans after the Second World War, as malnutrition during pregnancy leads to an effect on the health of the progeny in their adult life [1, 2]. It has already been well documented that suboptimal *in utero* environments in humans lead to long-term consequences for gene expression, DNA methylation, and phenotype [3]. These consequences of prenatal factors acting on the developing fetus are referred to as fetal programming [4]. In rodent models, nutrient-deficient prenatal diets have been widely studied with different modifications in terms of regimen type, time of exposure, and so on [5]. Our previous study applied a calorie-restricted diet [6], which was found to have greater impact on the lipid profile in the F0 dams than in their progeny. The hepatic expression level of the key lipid metabolism gene (*Fasn*) was also altered, while its methylation remained unchanged in response to the applied diet. This is in agreement with the earlier study of Lee et al. [7], in which the hepatic *Fasn* mRNA level also increased in response to the maternal calorie-restricted diet.

Early programmed changes during prenatal life may have consequences not only in adulthood, but can also be transmitted to the next generation. Such transgenerational inheritance has already been studied for calorie-restricted diets and their effect on the appetite [5]. The authors of that paper performed a meta-analysis of 35 studies in rats and mice, concluding that the transgenerational effects of restricted diets during pregnancy on progeny appetite were not significant, but that the reduced protein content in the diet significantly increased food intake in female offspring. Moreover, a comparison of obesogenic versus low-nutrition (protein-deficient) prenatal diets in terms of diabetes risk in the F2 generation was recently made by Hanafi et al. [8], who found that malnutrition had a greater transgenerational effect on F2, where glucose homeostasis was significantly altered simultaneously with overexpression of the glucose-sensing genes in fetal tissues. In a previous prenatal calorie-restricted diet study, we found changes in the expression profile of genes involved in epigenetic mechanisms (such as *Dnmt1*, *Mecp2*, *Hdac1*, and *Sin3a*), but the differences in the three generations were observed in the case of two genes [9]. Thus, it should be taken into account that the food deficiency can have an effect on DNA methylation and on histone modification profiles. Several studies have shown that modifications of epigenotype can be one of the mechanisms linking prenatal exposures and metabolism later in life [4].

In this study, we thus hypothesized that the use of a calorie-restricted diet during pregnancy in the rat can induce changes in hepatic DNA methylation and consequently lead to alterations in gene expression. To exclude the effect of postnatal feeding factors we performed the analysis for female fetuses at the age of 19^th^ days. Moreover, we assumed that the differences programmed in F1 can be inherited by the next generation. The aim of this study was to determine the effects of prenatal restricted diet on DNA methylation profile, as well as the transcript level of selected genes in the liver, in the female progeny of three generations of rats.

## Material and methods

### Animals and diets

The experiment was performed with the approval of the Bioethical Commission for Animal Care and Use in Poznan, Poland (approval no. 37/2014). The detailed procedures we followed have been described in our previous work [9]. Briefly, Wistar rats aged ten weeks were mated, with success confirmed by the presence of a vaginal plug. Half of the pregnant females were assigned to group R (restricted), receiving the experimental diet (50% of the typical food intake of the control (C) group, corrected for body mass), while group C received standard AIN-93G diet *ad libitum* [10]. At day 19 of pregnancy, a minimum of six pregnant dams in the C and R groups were anesthetized by CO_2_ inhalation and euthanized by cardiac puncture. The fetuses were euthanized by decapitation and the liver samples were collected, frozen, in liquid nitrogen, and stored at -80°C for further analysis. The remaining pregnant females were allowed to deliver and, after that, and in the following generations, all animals were fed standard AIN-93G diet *ad libitum*. To obtain the second generation, the F1 female offspring were kept until 8–10 weeks old and then mated with males. On day 19 of pregnancy, half of the pregnant dams were sacrificed to obtain the fetus livers from the second generation. The same procedure was replicated to obtain the third generation. Each studied group was composed from 6 random animals representing different litters. Body weight of the fetuses was measured as described earlier by Chmurzynska et al. [11] and was significantly reduced only in F1.

### Targeted bisulfite next generation sequencing

Based on the gene ontology (Rat Genome Database: https://rgd.mcw.edu/), we selected 1748 genes involved in six processes, namely: lipid metabolism, carbohydrate metabolism, energy homeostasis, methionine metabolism, mitochondria functioning, and imprinted genes. The total region of analysis spans over 2.1 Mb. Target regions were designed to overlap by approximately 1000 bp (800 bp upstream and 200 bp downstream) around the transcription start sites, which were determined on the basis of Ensembl 91 according to rat genome version rn6/RGSC6.0 (S1 File). Targeted bisulfite sequencing was performed using custom-designed sequence capture probes (SeqCap Epi Enrichment System, NimbleGen, Roche). The analysis was performed on twelve samples (six C and six R animals) of liver tissue derived from the F1 fetuses. Briefly, 1 μg of DNA was sonicated using Bioruptor (Diagenode) to obtain fragments of 180–220 bp following end repair, A-tailing, and adapter ligation with the use of KAPA Library Preparation Kit (KAPA Biosystems), according to the manufacturer’s protocol. Next, the bisulfite conversion was performed using EZ DNA Methylation Lightning Kit (Zymo Research), and enrichment by amplification was performed (SeqCap Epi Accessory kit, NimbleGene). The next step was hybridization for 72 h at 47°C with probes designed for the targeted genes (SeqCap Epi Developer, NimbleGene), following separation of the captured regions using SeqCap EZ Pure Capture Bead Kit (Roche). Finally, the libraries were enriched using a SeqCap Epi Accessory Kit (NimbleGene). Each library was composed of three individuals by multiplexing. The quality of the libraries, as well as the size distribution, was determined with the use of an Agilent DNA 1000 Kit on a 2100 Bioanalyzer (Agilent). The quantification of the library was measured on a Qubit 2.0 fluorometer (ThermoFisher Scientific). The final concentration was established to 12 pmol with a 20% contribution of PhiX genome DNA (Illumina) as an internal control. The sequencing runs were performed using a MiSeq Reagent Kit version 2, 2×150 bp (Illumina) on the MiSeq platform (Illumina), according to the manufacturer’s protocol. Reads were trimmed using Trimmomatic 0.36 software (https://www.ncbi.nlm.nih.gov/pmc/articles/PMC4103590/) with a sliding window algorithm. Mapping was performed with the use of Bismark 18.1 (https://www.ncbi.nlm.nih.gov/pubmed/21493656). Methylation levels were determined using MethylKit 1.3.8 (https://www.ncbi.nlm.nih.gov/pubmed/23034086). The raw NGS data are available from the European Nucleotide Archive (accession no: PRJEB26508).

### Validation of DNA methylation by pyrosequencing

DNA methylation analysis was performed by pyrosequencing for the seven CpG islands (CGIs) where the NGS data had identified at least one cytosine with a minimum difference of 10% in methylation levels between the C and R groups. These CGIs were located near or within genes such as *Usp30*, *Grb10*, *Pld1*, *St6galnac5*, *Oxct2b*, *Khk*, and *Acsl4*. DNA was isolated using the Genomic Mini DNA isolation kit (A&A Biotechnology) and the 500 ng was bisulfite-converted using EZ DNA Methylation Kit (Zymo Research). PCR amplification (with the use of biotinylated reverse primer at the 5’ end) of the studied fragments was performed using PyroMark PCR Kit (Qiagene) following 1.5% agarose gel electrophoresis to check the amplicon length. Next, pyrosequencing was conducted with the use of a PyroMark Q48 Advanced CpG Kit (Qiagene) on the PyroMark Q48 Autoprep System (Qiagene), according to the manufacturer’s protocol. This part was performed in liver samples derived from female fetuses from three generations (n = 6 per group). Details concerning the location of the examined fragments and primer sequences are given in Table A in S2 File.

### Relative transcript level by real-time PCR

The transcript levels of the seven genes (*Usp30*, *Grb10*, *Pld1*, *St6galnac5*, *Oxct2b*, *Khk*, and *Acsl4*) were determined in the liver of the three generations of rats from the restricted and control groups. Each generation/diet group was composed of six female fetuses from day 19 of pregnancy. The total RNA was isolated using Trizol (Roche) reagent, following the standard protocol. The quality and quantity of RNA were checked on a Nanodrop spectrophotometer. Reverse transcription was performed using High Fidelity cDNA Synthesis Kit (Roche) following real-time PCR on a Light Cycler 480 Instrument (Roche) using SYBR Green detection format (Roche). Each sample was analyzed in duplicate and the results were normalized using two reference genes—*Hprt* (hypoxanthine-guanine phosphoribosyltransferase) and *Tbp* (TATA box binding protein)—while relative transcript quantification was performed using the 2-ΔΔCT method, according to Livak and Schmittgen [12]. The details of the primer sequences and amplicon lengths are given in Table B in S2 File.

### Statistical and bioinformatics analyses

Using MethylKit, we selected only those cytosines with a coverage depth of at least ten reads in each of the samples. Additionally, positions with coverage higher than the 99.9th percentile were excluded. Differences in methylation levels with statistical significances were calculated according to the MethylKit user guide, using logistic regression and the SLIM method for adjusting p-values for multiple testing. Pathway clustering analysis of genes with statistically significant differences in methylation levels were performed with DAVID 6.8 [13] using KEGG pathways and the Bonferroni correction to adjust for multiple tests. The statistical significance of methylation level differences from pyrosequencing were determined using the two-tailed form of Student’s *t*-test. To determine the effect of generation on methylation levels within groups, based on pyrosequencing results, for each individual cytosine one way ANOVA from statsmodels 0.9 (http://www.statsmodels.org) was used, with the Bonferroni correction applied. Post-hoc analyses were conducted with the use of Tukey Honest Significant Differences test with family-wise error rate set to 0.05. Between-group differences in relative transcript levels were tested using one-way ANOVA. The data was analyzed using Statistica software (Statsoft, Tulsa, OK, USA).

## Results

### Targeted bisulfite NGS approach

On average, 75% of the reads successfully underwent the trimming procedure. Of this group, 25% of reads mapped to the region captured by molecular probes with an average depth coverage of 100 reads and more than 85% of region being covered by at least 40 reads. Conversion efficiency at least 99% across all samples.

**Fig 1:**
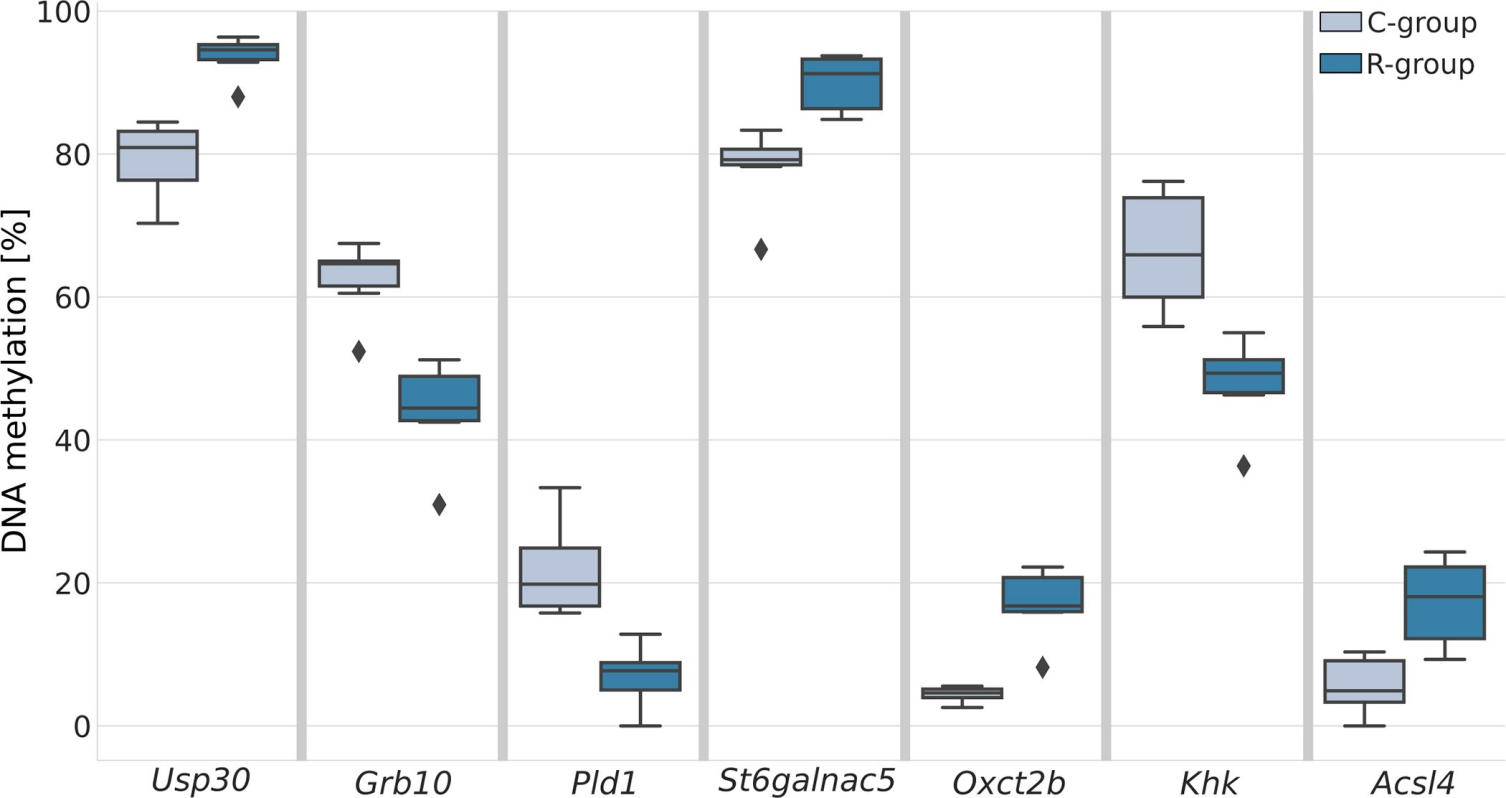
DNA methylation differences (≥ 10%) between the control and restricted groups. Results from targeted bisulfite next-generation sequencing of 1748 genes in the fetal liver. The boxes indicate the lower and upper quartiles, with the median marked by the horizontal line. Whiskers indicate 1.5 of the interquartile range. Outlying observations are marked with diamonds.

After rejecting cytosines with low coverage, we examined the methylation level differences in 108,019 positions located in 3458 CGIs. We obtained statistically significant differences in 394 positions (adjusted p-value (q-value) < 0.05) in close proximity to 374 genes. Ninety-eight percent of the indicated differences were lower than 10% (Figure A in S2 File). Gene clustering analyses, based on KEGG, revealed 23 significantly enriched pathways (Table C in S2 File). More detailed analysis indicated that among others, 22 genes are involved in oxidative phosphorylation processes (KEGG pathway ID: rno00190) associated with five mitochondrial membrane complexes (Table D in S2 File). However, the differences between the C and the R groups in the methylation level of cytosines varied from -3% to +3%. Thus, according to manufacturer’s recommendations, only seven cytosines which showed a difference of more than 10% between the studied groups (Fig 1) were selected for further analysis. They were located in the proximity of the *Usp30* (q-value = 0.0009), *Grb10* (q-value = 0.0285), *Pld1* (q-value = 0.0324), *St6galnac5* (q-value = 0.0255), *Oxct2b* (q-value = 0.0003), *Khk* (q-value = 0.0285), and *Acsl4* (q-value = 0.0480). The mean read’s coverage from the tNGS data for these selected cytosines reached from 33.5 for *Pld1* gene to 72.3 for *Oxct2b* gene (Table E in S2 File). Moreover, we also checked to which molecular pathways these genes have been assigned (according to KEGG database)–Table F in S2 File.

### Transgenerational DNA methylation analysis

**Fig 2:**
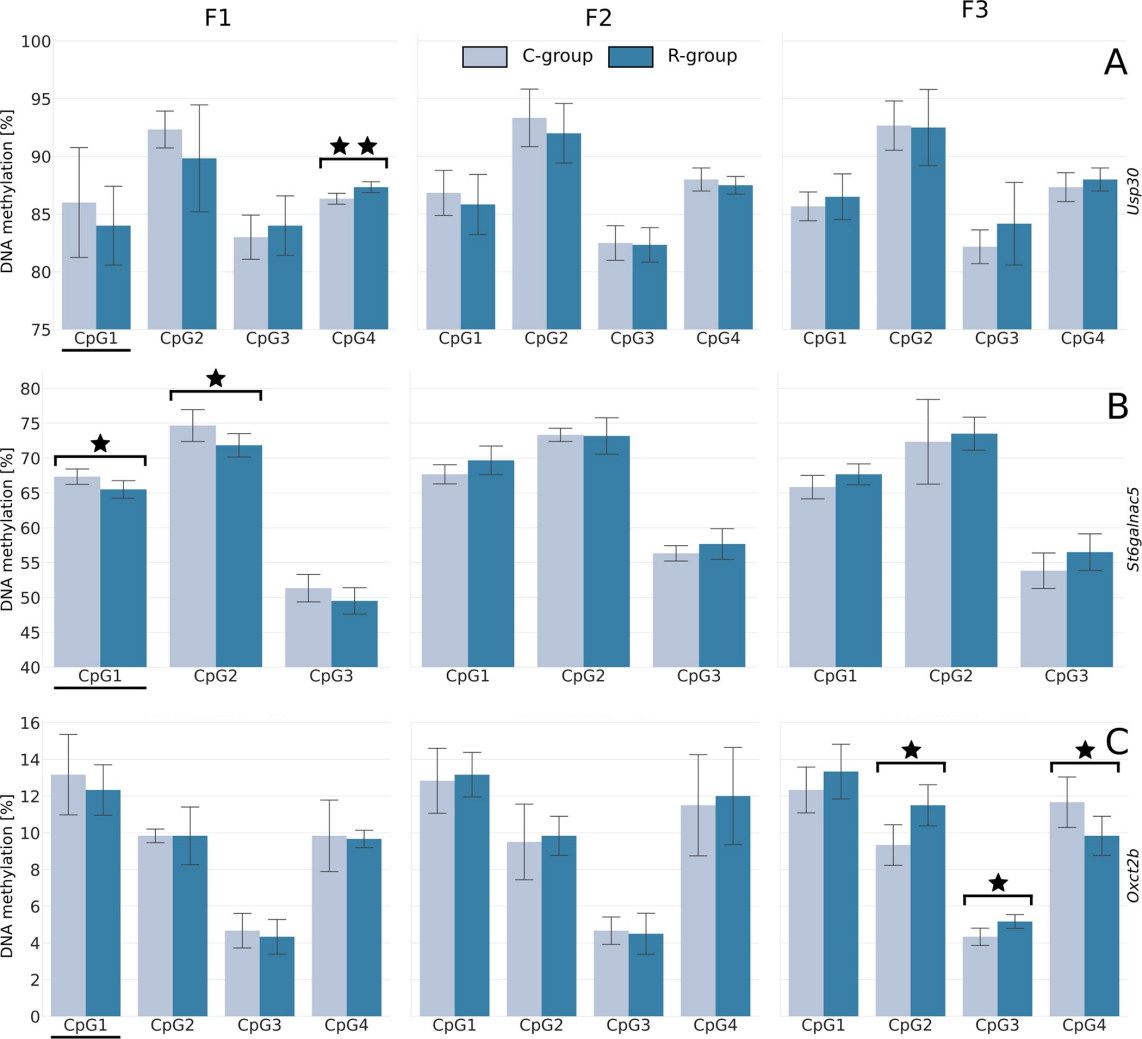
Transgenerational study of the cytosines (CpG) methylation levels by pyrosequencing. The underlined CpGs are these selected from NGS, while the nonunderlined CpGs are the adjacent to the CpG of interest. Values are means ± SDs. A: *Usp30*; B: *St6galnac5*; C: *Oxct2b* genes. A single star indicates significant differences: p = 0.034 for CpG1 and p = 0.049 for CpG2 in *St6galnac5*; p = 0.011 for CpG2, p = 0.011 for CpG3, and p = 0.040 for CpG4 in *Oxct2b*. A double star means p = 0.008 for CpG4 in *Usp30*.

The hepatic level of the seven cytosines selected from NGS was next determined in three generations of female fetuses. For all the studied cytosines, with the exception of the *Grb10* gene (where it was not possible to design primers near the cytosine), the amplicons covered the cytosine indicated by NGS, as well as the neighboring cytosines in the CGI. For *Grb10*, the sequenced fragment starts 217 bp upstream of the selected cytosine. The results showed that only two of the studied genes in F1—*Usp30* (CpG4, p = 0.008—Fig 2A) and *St6galnac5* (CpG1, p = 0.034 and CpG2, p = 0.049—Fig 2B) maintained a significant difference between the study groups, but in the case of the *St6galnac5* gene, the observed differences had the inverse proportions (with a decreased level in the restricted group). There were no differences in F2, while in F3 only the cytosines near *Oxct2b* significantly differed between the C and R groups (CpG2, p = 0.011; CpG3, p = 0.011 and CpG4, p = 0.040—Fig 2C). For *Grb10*, *Pld1*, *Khk*, and *Acsl4*, no difference was observed in any of the studied generations (Figure B in S2 File). Moreover, we performed additional analysis to check whether the level of methylation differed between generations independently from the applied dietary regimen and found such differences for *Grb10* and *St6galnac5* genes. The Tukey test showed that in all indicated cytosines the methylation level was lower in F1 generation as compared to F2 and F3, but there was no dietary group differences (Table G and Figure C in S2 File).

### Gene expression patterns

The hepatic transcript levels of the seven selected genes were established in the three generations. No differences were found in the F1 progeny for any of the genes. In F2, only the *Pld1* gene had a significantly (p = 0.0295; ε^2^ = 0.67) reduced mRNA level in the R group as compared to the controls, while in F3, *Oxct2b* showed increased (p = 0.0253; ε^2^ = 0.55) transcript levels in animals from the restricted group (Figure D in S2 File).

## Discussion

We performed targeted bisulfite NGS to determine the methylation level of over 1700 genes mostly involved in energy homeostasis pathways, and we found that over 300 cytosines showed very small though significant differences between the control and restricted groups. The clustering analysis indicated 23 detailed molecular pathways involving these genes. In the majority of these pathways, these genes were not directly functionally related to each other, with the exception of the oxidative phosphorylation process. In this pathway, 22 genes had altered DNA methylation—those encoding enzymes for NADH dehydrogenases, succinate dehydrogenases, ubiquinol-cytochrome c reductases, cytochrome c oxidases, pyrophosphatase, and ATP synthases. The differences were small (± 3%), but it cannot be excluded that such minor differences may affect metabolism, especially when they are involved in the same processes. The oxidative phosphorylation process that takes place in mitochondria is a key pathway where adenosine triphosphate (ATP) is produced as the main source of energy for cell functioning, as well as for the maintenance of metabolic homeostasis [14]. It can thus be speculated that altered methylation of genes involved in this process can change their expression levels, leading to a disruption of energy balance. Since small differences in a majority of genes were observed for other metabolic pathways, we anticipated that the use of the restricted diet during pregnancy did not dramatically alter DNA methylation in the offspring. In a previous study by Ogawa et al. [15], it was found that a prenatally experience calorie-restricted diet induced a wide range of changes in the hepatic methylome in the fetal liver of mice. It should be mentioned that Ogawa et al. used a different experimental approach in which the biological samples from individual animals were pooled and the methylation level was established using methylated DNA immunoprecipitation with microarray detection (Me-Dip-ChiP). They found that multiple genes involved in glucocorticoid signaling, insulin resistance, PPAR targets, cholesterol and fatty acid metabolism pathways, and immune response processes had altered methylation levels in their promoter regions. Of these, the imprinted *Grb10* gene (*growth factor receptor-bound protein 10*), involved in insulin and mTOR signaling pathways, was changed by the restricted maternal diet. This was also seen in our study, where NGS analysis showed reduced methylation in the restricted group. Ogawa et al. [15] also observed methylation differences in the *Acsl6* (long-chain-fatty-acid-CoA ligase 6) gene, which is involved in fatty acid metabolism, whereas our NGS study found such differences in *Acsl4*, which belongs to the same gene family. Another study employing a 50% reduction in the calorie intake of pregnant mice was performed by Ganguly et al. [16]. To determine the effect of the pregnant dams’ diet on the developing fetuses, the authors focused on transplacental glucose transport processes and studied the *Glut1* and *Glut3* genes. They noted that, in the restricted group, the methylation level in the promoter region of *Glut3* was significantly increased. Moreover, this led to enhanced binding of methyl-CpG-binding protein (Mecp2), together with histone deacetylases (Hdac2); consequently, a reduced level of placental transcription of *Glut3* gene was observed. This demonstrates that gene expression was altered by changed epigenetic mechanisms induced by the dietary factor. In our earlier study, we did not observe any such correlation for *Fasn*, a key lipid metabolism gene [6]. It should be mentioned that, in this study, the seven differently methylated cytosines indicated by NGS were also analyzed by pyrosequencing, and only one of them (near the *St6galnac5* gene) had such a correlation, though with a very small difference between the studied groups. This shows that the technological approach must be considered when drawing conclusions. Since minor effects on DNA methylation could be expected from the experimental procedures, the targeted methods applied here for mainly energy homeostasis genes should be extended to global DNA methylation analysis in the future. Moreover, we found that the methylation level for some cytosines in *Grb10* and *St6galnac5* genes in the first generation was lower than in F2 and F3, in both the C and R groups. A similar tendency, though not significant, was observed for the other examined genes. It can thus be speculated that unknown factors acting on both control and restricted groups affected the methylation profile in F1.

The DNA methylation results obtained here are consistent with our previous results on one-carbon metabolism, in which we analyzed the mothers of the fetuses examined in this work [11]. We showed that food deprivation leads to reduced S-adenosylmethionine concentrations in pregnant dams, but that methylation potential did not differ between the control and restricted groups. Moreover, the transcript levels of the genes from the one-carbon metabolism pathway were not altered.

It seems that a prenatal calorie-restricted diet acts more effectively on the gene expression profile than on epigenetic mechanisms. In the study of Ogawa et al. [15], the global hepatic expression profile, in response to the prenatal restricted diet, was established in mothers and fetuses using a microarray approach. Upregulation was observed in 1941 and 1335 genes in mothers and progeny, respectively. Slightly more genes were downregulated in the liver (2301 in the mothers and 1626 in the offspring). A 20% restriction in diet during the preconception and entire pregnancy periods in rat progeny was recently studied by Ramírez-López et al. [17]. They examined different tissue expression profiles and, in the liver of female offspring, found decreased expression levels of genes like *Pparα* and *Pparγ* (key genes involved in adipogenesis), *Acaca* and *Fasn* (which encode the *de novo* lipogenic enzymes), and *Insig1* and *Hmgcr* (important liver-specific cholesterol biosynthesis regulators). In consequence, the lipid profile in the female offspring was altered, with increased levels of plasma LDL concentration. This is in agreement with our previous data, where the LDL fraction was enhanced in the four-week old female offspring of mothers undergoing a 50% calorie restriction during pregnancy [6].

Transgenerational changes programmed by calorie-restricted diets have already been reported. Ponzio et al. [18] noted increased blood pressure in three generations of rats from the restricted group, while Araminaite et al. [19] found elevated body weight and shortened longevity in a male offspring of the F2 generation, indicating a sex-dependent effect of the maternal caloric restriction diet. Concerning the gene expression profile, Hoile et al. [20] employed a protein-restricted diet and concluded that only a tiny group of genes had the same expression profile in three generations, showing the transgenerational effect. Protein restriction was also studied by Burdge et al. [21], and a reduced level of DNA methylation in the promoter region of *Pparalfa* and *Gr* genes was seen in the F1 and F2 generations following elevated hepatic transcript level of these genes. In a study of mouse maternal undernutrition during the final week of pregnancy, changes in the expression of lipid and fatty acid metabolism genes were found in the F1 and F2 generations, while the transcript level of the imprinted genes was not altered [22]. In the present study, we found the test diet to have no effect on the hepatic transcript level of the seven studied genes in the first generation. It is thus unlikely that the differences observed in F2 and F3 were caused by the experimental diet. This is in agreement with our previous study [11], where no transgenerational effect was found in terms of hepatic one-carbon cycle metabolites and gene expression profiles. Interestingly, we observed that all the studied genes were overexpressed in the restricted group from F1, while they were underexpressed in F2. This suggests that other factors that affected gene expression might have occurred during F1 or F2 development.

## Conclusions

In summary, we postulate that use of the calorie-restricted diet during rat pregnancy did not dramatically alter the DNA methylation of the genes involved in energy homeostasis, and transgenerational inheritance was not observed. Moreover, changed CpG methylation was not accompanied by alterations in transcription level. However, the effect of some minor differences in the methylation level of strictly functionally related genes cannot be excluded. We speculate that overall food deficiency activates regulatory processes that optimize the intrauterine environment for the developing fetuses. These processes stimulate the use of stored nutrients and prevent deregulation of DNA methylation. It must be also borne in mind that we did not perform genome-wide methylation analysis, so changes in other groups of genes were possible.

## Supporting information

S1 File Supporting information (Table). (XLSX)

S2 File Supporting information (Tables and Figures). (DOCX)
