# Supplementary material for: Hepatic DNA methylation and expression profiles under prenatal restricted diet in three generations of female rat fetuses
Source: PLoS One. 2019 Apr 16;14(4):e0215471. doi: 10.1371/journal.pone.0215471 (PMC6467388; doi:10.1371/journal.pone.0215471)

**S2 File**

Supporting information

**Hepatic DNA methylation and expression profiles under prenatal restricted diet in three generations of female rat fetuses**

Joanna Nowacka-Woszuk,^1*^ Adrian Grzemski,^1^ Magdalena Sliwinska,^2^ Agata Chmurzynska^2^

^1^Department of Genetics and Animal Breeding, Poznan University of Life Sciences, Wolynska 33, 60-637 Poznan, Poland

^2^Institute of Human Nutrition and Dietetics, Poznan University of Life Sciences, Wojska Polskiego 31, 60-624 Poznan, Poland

^*^Corresponding author’s e-mail address: [joanna.nowacka-woszuk@up.poznan.pl](mailto:joanna.nowacka-woszuk@up.poznan.pl)

**Table A.** Location of the studied cytosines in the CpG islands, primers used in pyrosequencing, and amplicon lengths.

| Cytosines genomic location (rn6/RGSC6.0) | Location in a gene context | Primers sequences | amplicon length (bp) |
| --- | --- | --- | --- |
| 12:48316548 | 174 bp downstream of the *Usp30* | F: 5’ GGGTGGGAAAGTTTTGTAGTAAG  R: 5’ TTCTAAACCCACTCCAACCCCCTCAATCTA  seq: 5’ GGAGGTTATTTTGGTTATT | 178 |
| 14:92117731 | 192 bp downstream of the *Grb10* | F: 5’ AGTATAGGTGTTGGGAAGTTT  R: 5’ AAACTACAAACCCTATTCCTATTCTTACCC  seq: 5’ GGGAATTGATTGGGTAT | 327 |
| 2:113652068 | 280 bp upstream of the *Pld1* | F: 5’ AGGGATTTGTAAGAAGGTTATAGT  R: 5’ ACCTTCCTCCATACCTCAATTCTAA  seq: 5’ AGTTGGTTTTTTGTTGT | 293 |
| 2:258052775 | 60 bp downstream of the *St6galnac5* | F: 5’ TTTTTGGATGTTGGAATGTG  R: 5’ CCCCTACCTTACAACCCTTAAA  seq: 5’ GTGGGGGTTTTGTTGA | 206 |
| 5:140802879 | 759 bp upstream of the *Oxct2b* | F: 5’ AGTTTGTTTGGGGTTGGATTA  R: 5’ ATCACCAAAAACTACCAATACTAAAC  seq: 5’ GTTTTAGAGGAAAGTTAATAGTT | 200 |
| 6:26821801 | 842 bp upstream of the *Khk* | F: 5’ GTGGTAGGATGAGGTTGAAGATTTTTAGA  R: 5’ AACCACCCTCTCCCTTCCTAACTATCAT  seq: 5’ AGTTTTTAGAGTTTATG | 256 |
| X:113660107 | 163 bp upstream of the *Acsl4* | F: 5’ GGGGGAGTATAGTGGTGTG  R: 5’ ACCCCCCAAAAAACTTTCATCCTAAAT  seq: 5’ GTTAAGGAAGTTATTATTAAAGAGG | 330 |

**Table B.** Sequences of primers used for real-time PCR studies.

| Gene | Primers | Amplicon length |
| --- | --- | --- |
| *Usp30* | F: 5’ TGGGGTCCCATTACAGAGAG  R: 5’ TCAGCAGTGTCAAGGACAGG | 208 |
| *Grb10* | F: 5’ GATGGGACAAGCAAAGTGGT  R: 5’ ACGATCTCATGGTCCTCCAG | 164 |
| *Pld1* | F: 5’ ATCTACCTCTCTGGCTGTCCTG  R: 5’ GTATGTCTTTTGGTGGGAATGG | 220 |
| *St6galnac5* | F: 5’ CAACACTTGGCTGAGCACTG  R: 5’ TTGTACATTCATCGGGTCCA | 164 |
| *Oxct2b* | F: 5’ GAGCACTGCACCAAGACAAA  R: 5’ CCTTAACCTCGTCCAACGAC | 175 |
| *Khk* | F: 5’ TTCGGCTATGGAGAGGTGGT  R: 5’ TGAGCGTAGCCCCTTTCTTC | 118 |
| *Acsl4* | F: 5’ AGCCGCACTGAAGAATTGTC  R: 5’ TCTACCCCCTTCTGTTGTGC | 233 |

**Table C.** Clustering analysis into functional pathways of the 374 genes with significantly differed cytosines (accorging to KEGG database).

| **Pathway** | **No of genes** | **P value** | **P value (after Bonferroni correction)** | **Gene’s ID** |
| --- | --- | --- | --- | --- |
| rno01100: Metabolic pathways | 144 | 1.32306E-44 | 3.09595137598173E-42 | ENSRNOG00000000466, ENSRNOG00000005512, ENSRNOG00000002781, ENSRNOG00000010626, ENSRNOG00000024629, ENSRNOG00000019180, ENSRNOG00000001344, ENSRNOG00000019372, ENSRNOG00000048320, ENSRNOG00000011949, ENSRNOG00000014365, ENSRNOG00000001992, ENSRNOG00000014856, ENSRNOG00000001796, ENSRNOG00000009345, ENSRNOG00000049912, ENSRNOG00000017087, ENSRNOG00000032908, ENSRNOG00000017571, ENSRNOG00000008099, ENSRNOG00000012055, ENSRNOG00000046345, ENSRNOG00000021177, ENSRNOG00000010633, ENSRNOG00000013816, ENSRNOG00000005668, ENSRNOG00000008209, ENSRNOG00000009157, ENSRNOG00000003223, ENSRNOG00000009889, ENSRNOG00000019804, ENSRNOG00000016776, ENSRNOG00000003070, ENSRNOG00000006388, ENSRNOG00000001701, ENSRNOG00000007967, ENSRNOG00000012210, ENSRNOG00000010993, ENSRNOG00000014508, ENSRNOG00000012616, ENSRNOG00000017291, ENSRNOG00000028292, ENSRNOG00000020479, ENSRNOG00000028156, ENSRNOG00000021265, ENSRNOG00000059453, ENSRNOG00000018077, ENSRNOG00000005993, ENSRNOG00000014511, ENSRNOG00000011366, ENSRNOG00000017817, ENSRNOG00000032134, ENSRNOG00000012698, ENSRNOG00000012559, ENSRNOG00000010881, ENSRNOG00000011419, ENSRNOG00000003114, ENSRNOG00000007951, ENSRNOG00000029280, ENSRNOG00000016214, ENSRNOG00000038951, ENSRNOG00000046864, ENSRNOG00000004027, ENSRNOG00000024112, ENSRNOG00000026646, ENSRNOG00000018281, ENSRNOG00000042359, ENSRNOG00000000257, ENSRNOG00000029528, ENSRNOG00000005358, ENSRNOG00000018816, ENSRNOG00000003554, ENSRNOG00000006369, ENSRNOG00000008945, ENSRNOG00000008047, ENSRNOG00000000168, ENSRNOG00000026705, ENSRNOG00000015859, ENSRNOG00000006947, ENSRNOG00000008944, ENSRNOG00000000437, ENSRNOG00000016791, ENSRNOG00000016989, ENSRNOG00000006116, ENSRNOG00000013331, ENSRNOG00000002636, ENSRNOG00000018764, ENSRNOG00000059622, ENSRNOG00000018507, ENSRNOG00000007446, ENSRNOG00000032297, ENSRNOG00000060045, ENSRNOG00000046515, ENSRNOG00000004234, ENSRNOG00000003653, ENSRNOG00000005424, ENSRNOG00000000177, ENSRNOG00000002755, ENSRNOG00000019843, ENSRNOG00000028368, ENSRNOG00000009630, ENSRNOG00000037229, ENSRNOG00000020902, ENSRNOG00000018367, ENSRNOG00000037225, ENSRNOG00000052704, ENSRNOG00000016470, ENSRNOG00000009980, ENSRNOG00000016643, ENSRNOG00000018516, ENSRNOG00000011922, ENSRNOG00000007735, ENSRNOG00000008163, ENSRNOG00000000800, ENSRNOG00000052070, ENSRNOG00000008569, ENSRNOG00000011528, ENSRNOG00000017777, ENSRNOG00000047708, ENSRNOG00000043377, ENSRNOG00000028717, ENSRNOG00000011329, ENSRNOG00000018319, ENSRNOG00000042503, ENSRNOG00000009994, ENSRNOG00000028711, ENSRNOG00000007775, ENSRNOG00000019979, ENSRNOG00000018129, ENSRNOG00000001440, ENSRNOG00000021886, ENSRNOG00000017593, ENSRNOG00000039859, ENSRNOG00000028047, ENSRNOG00000001736, ENSRNOG00000011053, ENSRNOG00000016122, ENSRNOG00000056954, ENSRNOG00000038085, ENSRNOG00000008475, ENSRNOG00000002642, ENSRNOG00000049110, ENSRNOG00000007583, ENSRNOG00000021150 |
| rno04910: Insulin signaling pathway | 28 | 1.98241E-13 | 4.63989E-11 | ENSRNOG00000021147, ENSRNOG00000006388, ENSRNOG00000052343, ENSRNOG00000021689, ENSRNOG00000011377, ENSRNOG00000015480, ENSRNOG00000010957, ENSRNOG00000018494, ENSRNOG00000014366, ENSRNOG00000050578, ENSRNOG00000014904, ENSRNOG00000006116, ENSRNOG00000002833, ENSRNOG00000029986, ENSRNOG00000009079, ENSRNOG00000053869, ENSRNOG00000004060, ENSRNOG00000019228, ENSRNOG00000003463, ENSRNOG00000002946, ENSRNOG00000018903, ENSRNOG00000020902, ENSRNOG00000001868, ENSRNOG00000004978, ENSRNOG00000020657, ENSRNOG00000003919, ENSRNOG00000007583, ENSRNOG00000023428 |
| rno04932: Non-alcoholic fatty liver disease (NAFLD) | 27 | 4.56661E-11 | 1.06859E-08 | ENSRNOG00000005512, ENSRNOG00000018816, ENSRNOG00000001068, ENSRNOG00000007967, ENSRNOG00000008569, ENSRNOG00000020417, ENSRNOG00000048320, ENSRNOG00000011949, ENSRNOG00000028717, ENSRNOG00000013331, ENSRNOG00000002833, ENSRNOG00000042503, ENSRNOG00000029986, ENSRNOG00000018129, ENSRNOG00000017571, ENSRNOG00000019228, ENSRNOG00000003463, ENSRNOG00000021177, ENSRNOG00000017817, ENSRNOG00000032134, ENSRNOG00000002946, ENSRNOG00000018903, ENSRNOG00000005668, ENSRNOG00000026646, ENSRNOG00000016470, ENSRNOG00000018281, ENSRNOG00000023428 |
| rno00190: Oxidative phosphorylation | 24 | 7.0201E-10 | 1.6427E-07 | ENSRNOG00000005512, ENSRNOG00000017571, ENSRNOG00000008163, ENSRNOG00000018816, ENSRNOG00000008569, ENSRNOG00000007967, ENSRNOG00000021177, ENSRNOG00000017817, ENSRNOG00000000557, ENSRNOG00000032134, ENSRNOG00000048320, ENSRNOG00000011949, ENSRNOG00000005668, ENSRNOG00000028717, ENSRNOG00000038951, ENSRNOG00000001992, ENSRNOG00000052704, ENSRNOG00000013331, ENSRNOG00000016470, ENSRNOG00000026646, ENSRNOG00000042503, ENSRNOG00000018281, ENSRNOG00000049912, ENSRNOG00000018129 |
| rno04931: Insulin resistance | 20 | 5.92681E-09 | 1.38687E-06 | ENSRNOG00000053869, ENSRNOG00000042411, ENSRNOG00000006388, ENSRNOG00000019228, ENSRNOG00000021689, ENSRNOG00000003463, ENSRNOG00000015480, ENSRNOG00000018494, ENSRNOG00000002946, ENSRNOG00000018903, ENSRNOG00000020902, ENSRNOG00000014904, ENSRNOG00000019742, ENSRNOG00000002833, ENSRNOG00000003919, ENSRNOG00000029986, ENSRNOG00000018507, ENSRNOG00000007583, ENSRNOG00000023428, ENSRNOG00000019057 |
| rno00510: N-Glycan biosynthesis | 14 | 9.36456E-09 | 2.19131E-06 | ENSRNOG00000017087, ENSRNOG00000004234, ENSRNOG00000046515, ENSRNOG00000000800, ENSRNOG00000014511, ENSRNOG00000006369, ENSRNOG00000012055, ENSRNOG00000011528, ENSRNOG00000046345, ENSRNOG00000010993, ENSRNOG00000012559, ENSRNOG00000016989, ENSRNOG00000049110, ENSRNOG00000011922 |
| rno00310: Lysine degradation | 14 | 1.57418E-08 | 3.68358E-06 | ENSRNOG00000016680, ENSRNOG00000001417, ENSRNOG00000024629, ENSRNOG00000001344, ENSRNOG00000061499, ENSRNOG00000015621, ENSRNOG00000028207, ENSRNOG00000016790, ENSRNOG00000004027, ENSRNOG00000013045, ENSRNOG00000020386, ENSRNOG00000017508, ENSRNOG00000021614, ENSRNOG00000023317 |
| rno04150: mTOR signaling pathway | 15 | 1.80238E-08 | 4.21756E-06 | ENSRNOG00000042411, ENSRNOG00000052343, ENSRNOG00000019228, ENSRNOG00000021689, ENSRNOG00000037505, ENSRNOG00000002763, ENSRNOG00000010957, ENSRNOG00000050578, ENSRNOG00000018903, ENSRNOG00000012061, ENSRNOG00000057078, ENSRNOG00000017426, ENSRNOG00000017297, ENSRNOG00000003919, ENSRNOG00000023428 |
| rno00564: Glycerophospholipid metabolism | 17 | 1.40689E-07 | 3.29207E-05 | ENSRNOG00000018077, ENSRNOG00000008320, ENSRNOG00000000177, ENSRNOG00000025012, ENSRNOG00000026705, ENSRNOG00000000437, ENSRNOG00000028368, ENSRNOG00000016791, ENSRNOG00000009980, ENSRNOG00000014856, ENSRNOG00000024112, ENSRNOG00000028156, ENSRNOG00000018319, ENSRNOG00000016643, ENSRNOG00000012269, ENSRNOG00000021265, ENSRNOG00000001796 |
| rno05012: Parkinson's disease | 21 | 2.65611E-07 | 6.2151E-05 | ENSRNOG00000005512, ENSRNOG00000017571, ENSRNOG00000018816, ENSRNOG00000008569, ENSRNOG00000007967, ENSRNOG00000021177, ENSRNOG00000017817, ENSRNOG00000019465, ENSRNOG00000032134, ENSRNOG00000048320, ENSRNOG00000011949, ENSRNOG00000005668, ENSRNOG00000028717, ENSRNOG00000004978, ENSRNOG00000013331, ENSRNOG00000016470, ENSRNOG00000026646, ENSRNOG00000042503, ENSRNOG00000018281, ENSRNOG00000049912, ENSRNOG00000018129 |
| rno05010: Alzheimer's disease | 23 | 3.0069E-07 | 7.03589E-05 | ENSRNOG00000005512, ENSRNOG00000021147, ENSRNOG00000017571, ENSRNOG00000004060, ENSRNOG00000018816, ENSRNOG00000008569, ENSRNOG00000007967, ENSRNOG00000021177, ENSRNOG00000017817, ENSRNOG00000032134, ENSRNOG00000048320, ENSRNOG00000011949, ENSRNOG00000005668, ENSRNOG00000028717, ENSRNOG00000013331, ENSRNOG00000002833, ENSRNOG00000026646, ENSRNOG00000016470, ENSRNOG00000042503, ENSRNOG00000018281, ENSRNOG00000049912, ENSRNOG00000018129, ENSRNOG00000021150 |
| rno05231: Choline metabolism in cancer | 16 | 1.85099E-06 | 0.000433038 | ENSRNOG00000008320, ENSRNOG00000019228, ENSRNOG00000001068, ENSRNOG00000021689, ENSRNOG00000000177, ENSRNOG00000050578, ENSRNOG00000026705, ENSRNOG00000018903, ENSRNOG00000016791, ENSRNOG00000012061, ENSRNOG00000009980, ENSRNOG00000024112, ENSRNOG00000028156, ENSRNOG00000003919, ENSRNOG00000001796, ENSRNOG00000023428 |
| rno00600: Sphingolipid metabolism | 11 | 5.51419E-06 | 0.001289491 | ENSRNOG00000002781, ENSRNOG00000009980, ENSRNOG00000032942, ENSRNOG00000000177, ENSRNOG00000012210, ENSRNOG00000003223, ENSRNOG00000010626, ENSRNOG00000036866, ENSRNOG00000012953, ENSRNOG00000009345, ENSRNOG00000000257 |
| rno00514: Other types of O-glycan biosynthesis | 8 | 6.277E-06 | 0.001467745 | ENSRNOG00000019843, ENSRNOG00000046515, ENSRNOG00000050298, ENSRNOG00000012146, ENSRNOG00000028207, ENSRNOG00000010104, ENSRNOG00000001417, ENSRNOG00000023317 |
| rno05016: Huntington's disease | 22 | 7.18234E-06 | 0.001679261 | ENSRNOG00000005512, ENSRNOG00000017571, ENSRNOG00000018816, ENSRNOG00000008569, ENSRNOG00000000604, ENSRNOG00000007967, ENSRNOG00000019298, ENSRNOG00000021177, ENSRNOG00000017817, ENSRNOG00000032134, ENSRNOG00000048320, ENSRNOG00000011949, ENSRNOG00000005668, ENSRNOG00000028717, ENSRNOG00000013331, ENSRNOG00000016470, ENSRNOG00000026646, ENSRNOG00000042503, ENSRNOG00000018281, ENSRNOG00000049912, ENSRNOG00000018129, ENSRNOG00000021150 |
| rno04012: ErbB signaling pathway | 14 | 1.40695E-05 | 0.003286883 | ENSRNOG00000021147, ENSRNOG00000019228, ENSRNOG00000021689, ENSRNOG00000010957, ENSRNOG00000006450, ENSRNOG00000014366, ENSRNOG00000018903, ENSRNOG00000017879, ENSRNOG00000012061, ENSRNOG00000001868, ENSRNOG00000002833, ENSRNOG00000020657, ENSRNOG00000003919, ENSRNOG00000023428 |
| rno00534: Glycosaminoglycan biosynthesis - heparan sulfate / heparin | 8 | 1.61706E-05 | 0.003776809 | ENSRNOG00000016776, ENSRNOG00000017659, ENSRNOG00000024591, ENSRNOG00000010598, ENSRNOG00000021886, ENSRNOG00000019979, ENSRNOG00000019804, ENSRNOG00000008944 |
| rno04070: Phosphatidylinositol signaling system | 14 | 2.53482E-05 | 0.005913996 | ENSRNOG00000056954, ENSRNOG00000012061, ENSRNOG00000004060, ENSRNOG00000019228, ENSRNOG00000020479, ENSRNOG00000024112, ENSRNOG00000021265, ENSRNOG00000001796, ENSRNOG00000018516, ENSRNOG00000023428, ENSRNOG00000026705, ENSRNOG00000060045, ENSRNOG00000021150, ENSRNOG00000018903 |
| rno00563: Glycosylphosphatidylinositol(GPI)-anchor biosynthesis | 8 | 2.81363E-05 | 0.006562353 | ENSRNOG00000029280, ENSRNOG00000003070, ENSRNOG00000003554, ENSRNOG00000011366, ENSRNOG00000059622, ENSRNOG00000049110, ENSRNOG00000042359, ENSRNOG00000007735 |
| rno00561: Glycerolipid metabolism | 11 | 3.0989E-05 | 0.007225319 | ENSRNOG00000000437, ENSRNOG00000018077, ENSRNOG00000004027, ENSRNOG00000009980, ENSRNOG00000000177, ENSRNOG00000024112, ENSRNOG00000001796, ENSRNOG00000001344, ENSRNOG00000028711, ENSRNOG00000014508, ENSRNOG00000026705 |
| rno01130: Biosynthesis of antibiotics | 21 | 7.29922E-05 | 0.016935737 | ENSRNOG00000003653, ENSRNOG00000032908, ENSRNOG00000001440, ENSRNOG00000005424, ENSRNOG00000007967, ENSRNOG00000024629, ENSRNOG00000001344, ENSRNOG00000043377, ENSRNOG00000011329, ENSRNOG00000016122, ENSRNOG00000018367, ENSRNOG00000038085, ENSRNOG00000006116, ENSRNOG00000013331, ENSRNOG00000028292, ENSRNOG00000004027, ENSRNOG00000009994, ENSRNOG00000018507, ENSRNOG00000032297, ENSRNOG00000009889, ENSRNOG00000029528 |
| rno04152: AMPK signaling pathway | 15 | 0.000137029 | 0.031558398 | ENSRNOG00000019228, ENSRNOG00000021689, ENSRNOG00000003463, ENSRNOG00000010728, ENSRNOG00000050578, ENSRNOG00000018911, ENSRNOG00000018903, ENSRNOG00000016122, ENSRNOG00000013552, ENSRNOG00000020902, ENSRNOG00000005389, ENSRNOG00000017297, ENSRNOG00000003919, ENSRNOG00000029986, ENSRNOG00000023428 |
| rno04666: Fc gamma R-mediated phagocytosis | 12 | 0.00016187 | 0.037172188 | ENSRNOG00000012061, ENSRNOG00000001868, ENSRNOG00000019228, ENSRNOG00000001068, ENSRNOG00000021689, ENSRNOG00000009980, ENSRNOG00000028156, ENSRNOG00000000177, ENSRNOG00000003919, ENSRNOG00000010626, ENSRNOG00000023428, ENSRNOG00000018903 |

**Table D.** Genes involved in oxidative phosphorylation with altered cytosine methylation in R group when comparing to C group.

| **Mitochondrial membrane complex** | **Genes with altered cytosine methylation** | **Chromosome and cytosine position (rn6/RGSC6.0)** | **% of difference in methylation in R group comparing to C group** |
| --- | --- | --- | --- |
| I - NADH-coenzyme Q oxidoreductase | *Ndufa10* | 9:99651701 | 2.6415 |
|  | *Ndufa11* | 9:10338253 | 1.7341 |
|  | *Ndufa2* | 18:29587615 | -1.7483 |
|  | *Ndufa2* | 18:29587928 | 1.4493 |
|  | *Ndufa4* | 4:38241166 | -1.9520 |
|  | *Ndufa6* | 7:123586929 | 1.7263 |
|  | *Ndufa6* | 7:123586974 | -1.9820 |
|  | *Ndufa8* | 3:15379153 | -3.3846 |
|  | *Ndufab1* | 1:192057610 | 1.9727 |
|  | *Ndufb5* | 2:119139606 | -1.3447 |
|  | *Ndufb7* | 19:24701019 | 2.1622 |
|  | *Ndufs5* | 5:141405271 | 2.4540 |
|  | *Ndufv2* | 9:113900145 | -2.1638 |
|  | *Ndufv2* | 9:113900307 | -2.4433 |
| II - Succinate-Q oxidoreductase | *Sdha* | 1:31545326 | 1.2642 |
|  | *Sdhb* | 5:159484430 | 1.6000 |
| III - Q-cytochrome c oxidoreductase | *Uqcrc1* | 8:117679138 | 1.7568 |
|  | *Uqcrfs1* | 17:35678011 | 2.4272 |
| IV - Cytochrome c oxidase | *Cox17* | 11:64968378 | -3.2258 |
|  | *Cox4i1* | 19:54245885 | 2.1033 |
|  | *Cox5a* | 8:62298290 | -2.2312 |
|  | *Cox5a* | 8:62298415 | -2.7837 |
|  | *Cox8a* | 1:222468938 | 1.4742 |
| V - ATP synthase | *Ppa1* | 20:31265624 | 2.2409 |
|  | *Atp6v1a* | 11:61530667 | 3.0641 |
|  | *Atp6v1g1* | 5:79367706 | -2.6634 |

+ or – means that the methylation in R group was higher or lower than in C group, respectively

**Table E.** Mean coverage after tNGS (± SD) for the analysed cytosines in the seven selected genes

| **Gene** | **C-group** | | **R-group** | |
| --- | --- | --- | --- | --- |
|  | **Mean** | **± SD** | **Mean** | **± SD** |
| *Usp30* | 54.67 | 7.69 | 46.50 | 18.36 |
| *Grb10* | 40.83 | 4.75 | 40.00 | 9.94 |
| *Pld1* | 35.50 | 13.44 | 33.50 | 12.29 |
| *St6galnac5* | 54.00 | 8.07 | 51.17 | 14.86 |
| *Oxct2b* | 67.33 | 20.87 | 72.33 | 30.19 |
| *Khk* | 41.67 | 5.01 | 39.00 | 11.40 |
| *Acsl4* | 41.67 | 14.36 | 37.33 | 13.03 |

**Table F.** The assignment of the selected seven genes to particular KEGG pathways.

| **Gene** | **Pathways (rat database)** |
| --- | --- |
| ***Grb10*** *(growth factor receptor bound protein 10)* | rno04150: mTOR signalling pathway |
| ***Oxct2b*** *(3-oxoacid CoA transferase 2B)* | no pathway for rat |
| ***Usp30*** *(ubiquitin specific peptidase 30)* | rno04137: Mitophagy - animal |
| ***Pld1*** *(phospholipase D1)* | rno00564: Glycerophospholipid metabolism  rno00565: Ether lipid metabolism  rno01100: Metabolic pathways  rno04014: Ras signalling pathway  rno04024: cAMP signalling pathway  rno04071: Sphingolipid signalling pathway  rno04072: Phospholipase D signalling pathway  rno04144: Endocytosis  rno04666: Fc gamma R-mediated phagocytosis  rno04724: Glutamatergic synapse  rno04912: GnRH signalling pathway  rno04928: Parathyroid hormone synthesis, secretion and action  rno05212: Pancreatic cancer  rno05231: Choline metabolism in cancer |
| ***Khk*** *(ketohexokinase)* | rno00051: Fructose and mannose metabolism  rno01100: Metabolic pathways |
| ***St6galnac5*** *(ST6 N-acetylgalactosaminide alpha-2,6-sialyltransferase 5)* | rno00604: Glycosphingolipid biosynthesis - ganglio series  rno01100: Metabolic pathways |
| ***Acsl4*** *(acyl-CoA synthetase long-chain family member 4)* | rno00061: Fatty acid biosynthesis  rno00071: Fatty acid degradation  rno01100: Metabolic pathways  rno01212: Fatty acid metabolism  rno03320: PPAR signalling pathway  rno04146: Peroxisome  rno04216: Ferroptosis  rno04714: Thermogenesis  rno04920: Adipocytokine signalling pathway |

**Table G.** Methylation level analysis of particular cytosines across generations.

| **Gene** | **CpG** | **Group** | **P value** | **P value (after Bonferroni correction)** | **Tukey Honest Significant Test**  **(mean difference and S: significant or NS: not significant)** | | |
| --- | --- | --- | --- | --- | --- | --- | --- |
|  |  |  |  |  | **F1 versus F2** | **F1 versus F3** | **F2 versus F3** |
| *Grb10* | CpG1 | C | 0.2339 | 1.0000 | NA | NA | NA |
|  | CpG1 | R | 0.2888 | 1.0000 | NA | NA | NA |
|  | CpG2 | C | 0.3149 | 1.0000 | NA | NA | NA |
|  | CpG2 | R | 0.5150 | 1.0000 | NA | NA | NA |
|  | CpG3** | C | 0.0000 | 0.0005 | 13.833 (S) | 12.666 (S) | -1.166 (NS) |
|  | CpG3* | R | 0.0001 | 0.0036 | 12.500 (S) | 10.833 (S) | -1.666 (NS) |
|  | CpG4* | C | 0.0000 | 0.0019 | 14.500 (S) | 15.000 (S) | 0.500 (NS) |
|  | CpG4** | R | 0.0000 | 0.0000 | 16.333 (S) | 16.500 (S) | 0.166 (NS) |
|  | CpG5** | C | 0.0000 | 0.0003 | 15.333 (S) | 15.000 (S) | -0.333 (NS) |
|  | CpG5** | R | 0.0000 | 0.0000 | 16.333 (S) | 16.166 (S) | -0.166 (NS) |
| *Oxct2b* | CpG1 | C | 0.7613 | 1.0000 | NA | NA | NA |
|  | CpG1 | R | 0.4800 | 1.0000 | NA | NA | NA |
|  | CpG2 | C | 0.8426 | 1.0000 | NA | NA | NA |
|  | CpG2 | R | 0.0889 | 1.0000 | NA | NA | NA |
|  | CpG3 | C | 0.7217 | 1.0000 | NA | NA | NA |
|  | CpG3 | R | 0.3066 | 1.0000 | NA | NA | NA |
|  | CpG4 | C | 0.3398 | 1.0000 | NA | NA | NA |
|  | CpG4 | R | 0.0779 | 1.0000 | NA | NA | NA |
| *Usp30* | CpG1 | C | 0.8263 | 1.0000 | NA | NA | NA |
|  | CpG1 | R | 0.3512 | 1.0000 | NA | NA | NA |
|  | CpG2 | C | 0.7512 | 1.0000 | NA | NA | NA |
|  | CpG2 | R | 0.4797 | 1.0000 | NA | NA | NA |
|  | CpG3 | C | 0.7257 | 1.0000 | NA | NA | NA |
|  | CpG3 | R | 0.5072 | 1.0000 | NA | NA | NA |
|  | CpG4 | C | 0.0462 | 1.0000 | NA | NA | NA |
|  | CpG4 | R | 0.3911 | 1.0000 | NA | NA | NA |
| *Pld1* | CpG1 | C | 0.8036 | 1.0000 | NA | NA | NA |
|  | CpG1 | R | 0.0917 | 1.0000 | NA | NA | NA |
|  | CpG2 | C | 0.6844 | 1.0000 | NA | NA | NA |
|  | CpG2 | R | 0.9256 | 1.0000 | NA | NA | NA |
|  | CpG3 | C | 0.1609 | 1.0000 | NA | NA | NA |
|  | CpG3 | R | 0.2423 | 1.0000 | NA | NA | NA |
|  | CpG4 | C | 0.5425 | 1.0000 | NA | NA | NA |
|  | CpG4 | R | 0.0600 | 1.0000 | NA | NA | NA |
| *St6galnac5* | CpG1 | C | 0.1230 | 1.0000 | NA | NA | NA |
|  | CpG1 | R | 0.0041 | 0.2453 | NA | NA | NA |
|  | CpG2 | C | 0.6291 | 1.0000 | NA | NA | NA |
|  | CpG2 | R | 0.4814 | 1.0000 | NA | NA | NA |
|  | CpG3 | C | 0.0041 | 0.2478 | NA | NA | NA |
|  | CpG3* | R | 0.0001 | 0.0046 | 8.166 (S) | 7.000 (S) | -1.166 (NS) |
| *Khk* | CpG1 | C | 0.0575 | 1.0000 | NA | NA | NA |
|  | CpG1 | R | 0.2216 | 1.0000 | NA | NA | NA |
|  | CpG2 | C | 0.1402 | 1.0000 | NA | NA | NA |
|  | CpG2 | R | 0.0269 | 1.0000 | NA | NA | NA |
|  | CpG3 | C | 0.3828 | 1.0000 | NA | NA | NA |
|  | CpG3 | R | 0.1012 | 1.0000 | NA | NA | NA |
|  | CpG4 | C | 0.0431 | 1.0000 | NA | NA | NA |
|  | CpG4 | R | 0.1130 | 1.0000 | NA | NA | NA |
|  | CpG5 | C | 0.1042 | 1.0000 | NA | NA | NA |
|  | CpG5 | R | 0.5638 | 1.0000 | NA | NA | NA |
| *Acsl4* | CpG1 | C | 0.2444 | 1.0000 | NA | NA | NA |
|  | CpG1 | R | 0.0009 | 0.0540 | NA | NA | NA |
|  | CpG2 | C | 0.0143 | 0.8568 | NA | NA | NA |
|  | CpG2 | R | 0.0187 | 1.0000 | NA | NA | NA |
|  | CpG3 | C | 0.1625 | 1.0000 | NA | NA | NA |
|  | CpG3 | R | 0.0066 | 0.3974 | NA | NA | NA |
|  | CpG4 | C | 0.0785 | 1.0000 | NA | NA | NA |
|  | CpG4 | R | 0.7792 | 1.0000 | NA | NA | NA |
|  | CpG5 | C | 0.0518 | 1.0000 | NA | NA | NA |
|  | CpG5 | R | 0.0380 | 1.0000 | NA | NA | NA |

* p value < 0.05; ** p value < 0.001 after Bonferroni correction; NA-not analysed

**Figure A.** The histogram of absolute differences in methylation levels of cytosines, between studied groups from F1 fetuses. Y-axis indicates frequency of cytosines with detected absolute difference in methylation level on X-axis. Methylation levels differences are expressed as percentages. Only cytosines with absolute difference higher than 10% were selected for further analyses.


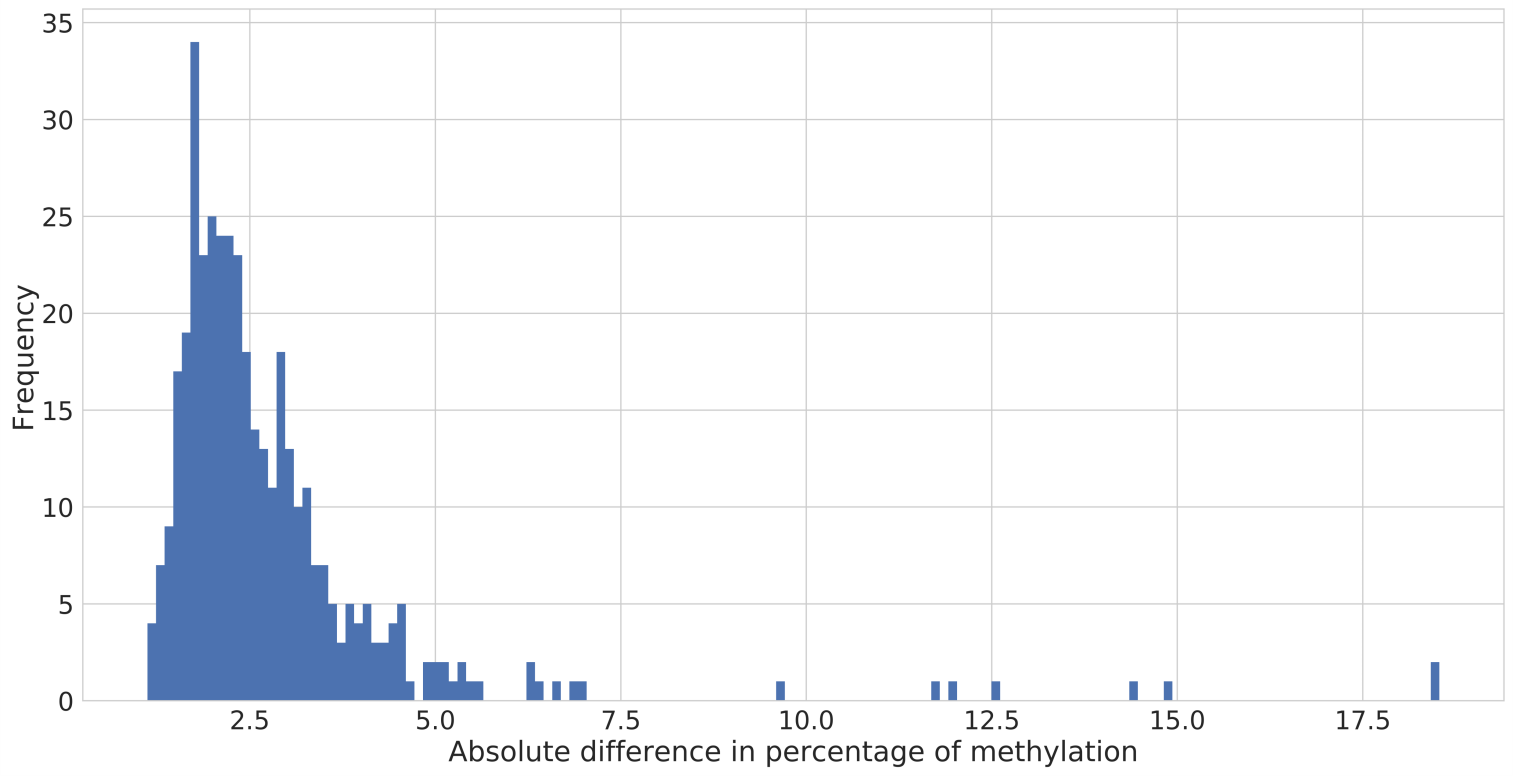


**Figure B.** Transgenerational study of cytosine (CpG) methylation levels by pyrosequencing. Values are means ± SDs. A: *Khk*; B: *Grb10*; C: *Pld1*; D: *Acsl4*. The underlined cytosines are those selected from NGS.


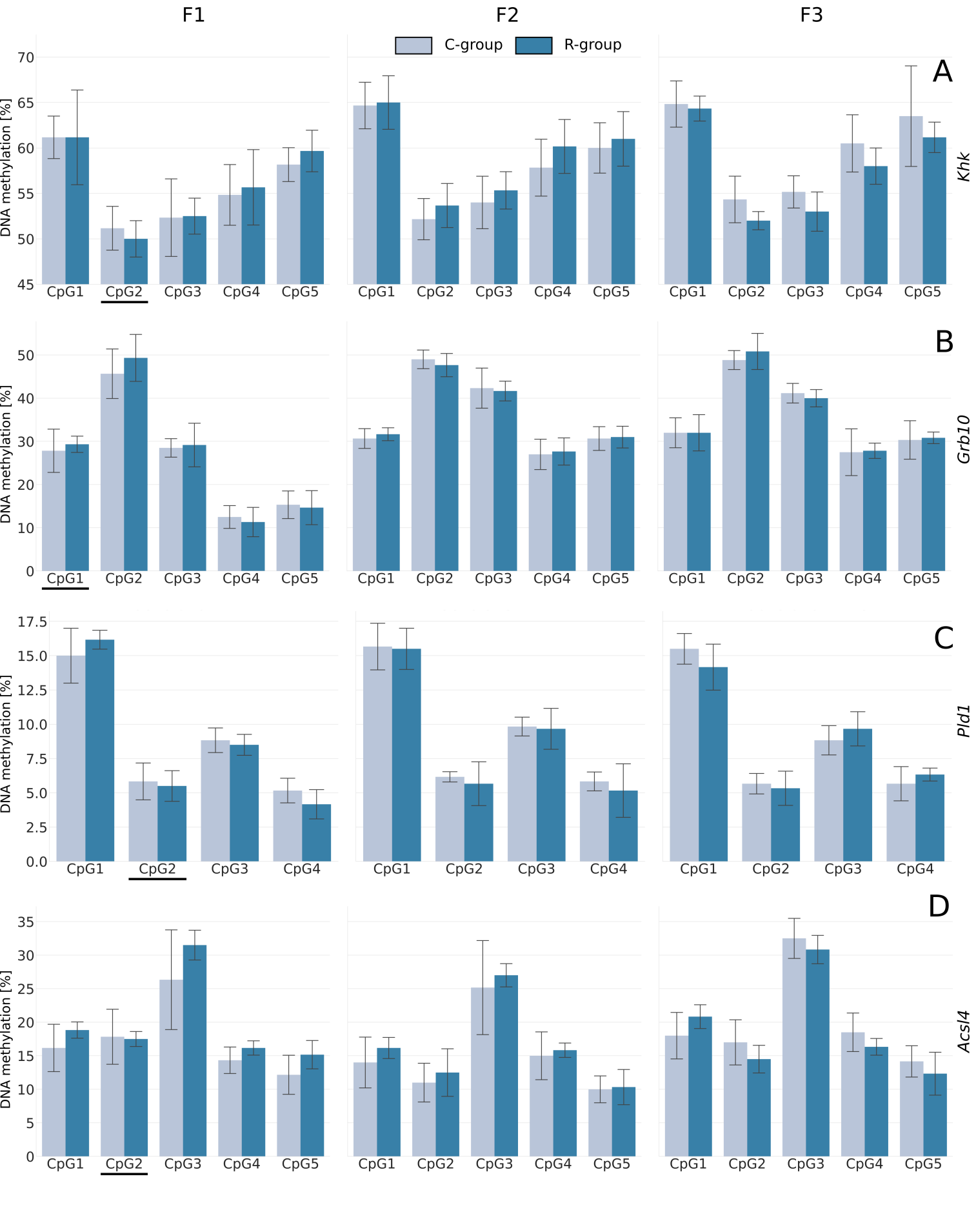


**Figure C.** The transgenerational analysis of methylation level across generation. The boxes indicate the lower and upper quartiles, with the median marked by the horizontal line. Whiskers indicate 1.5 of the interquartile range. Outlying observations are marked with diamonds. Ona-way ANOVA was applied to compare group means. A star and double star, indicate a significant difference of p < 0.05 and p < 0.001, respectively.


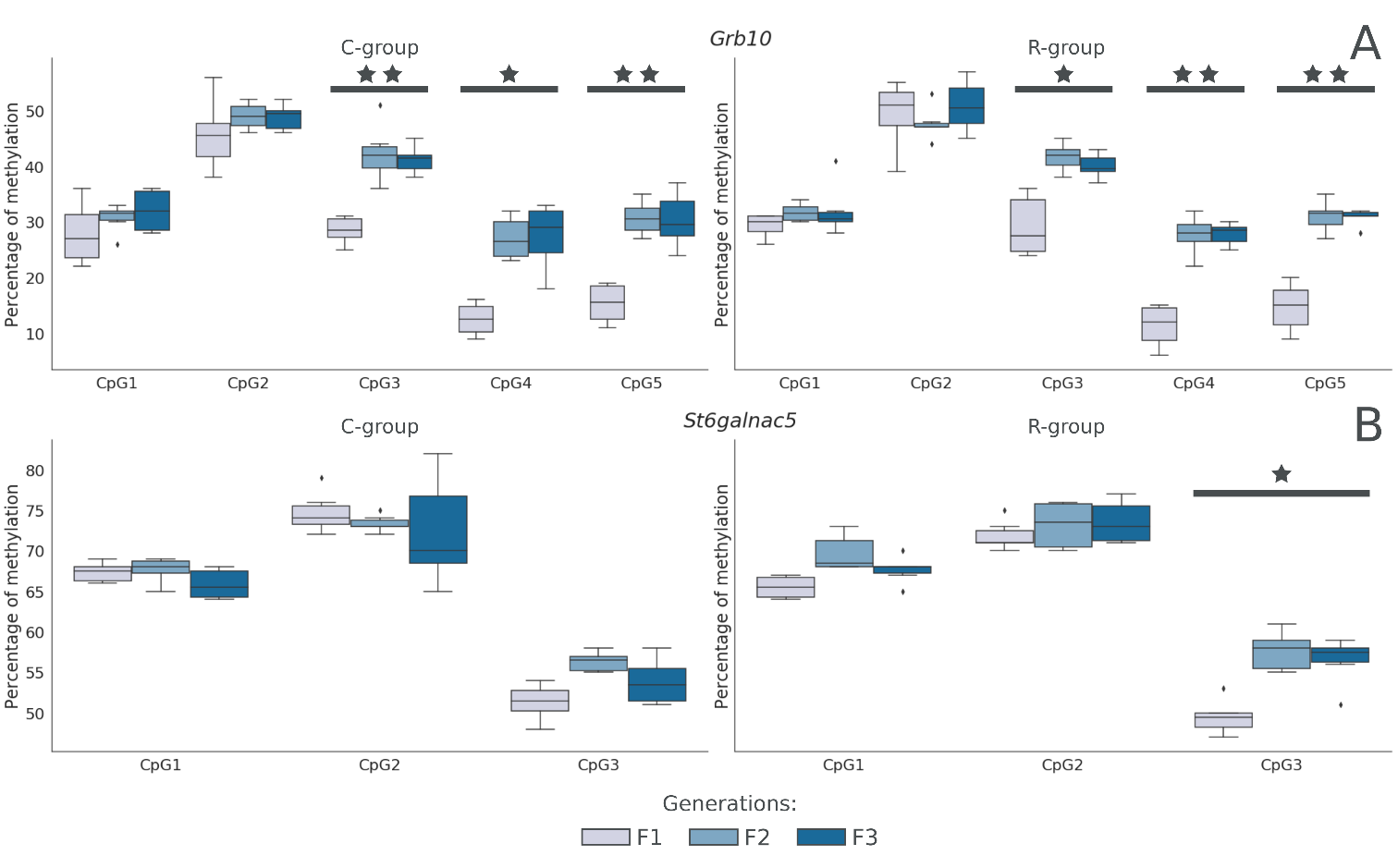


**Figure D.** The hepatic relative transcript level in three generations of rats. The results show the deltaCt value. The boxes indicate the lower and upper quartiles, with the median marked by the horizontal line. Whiskers indicate 1.5 of the interquartile range. Outlying observations are marked with diamonds. Ona-way ANOVA was applied to compare group means. A star indicates a significant difference (p = 0.0295 for *Pld1* and p = 0.0253 for *Oxct2b*, respectively).


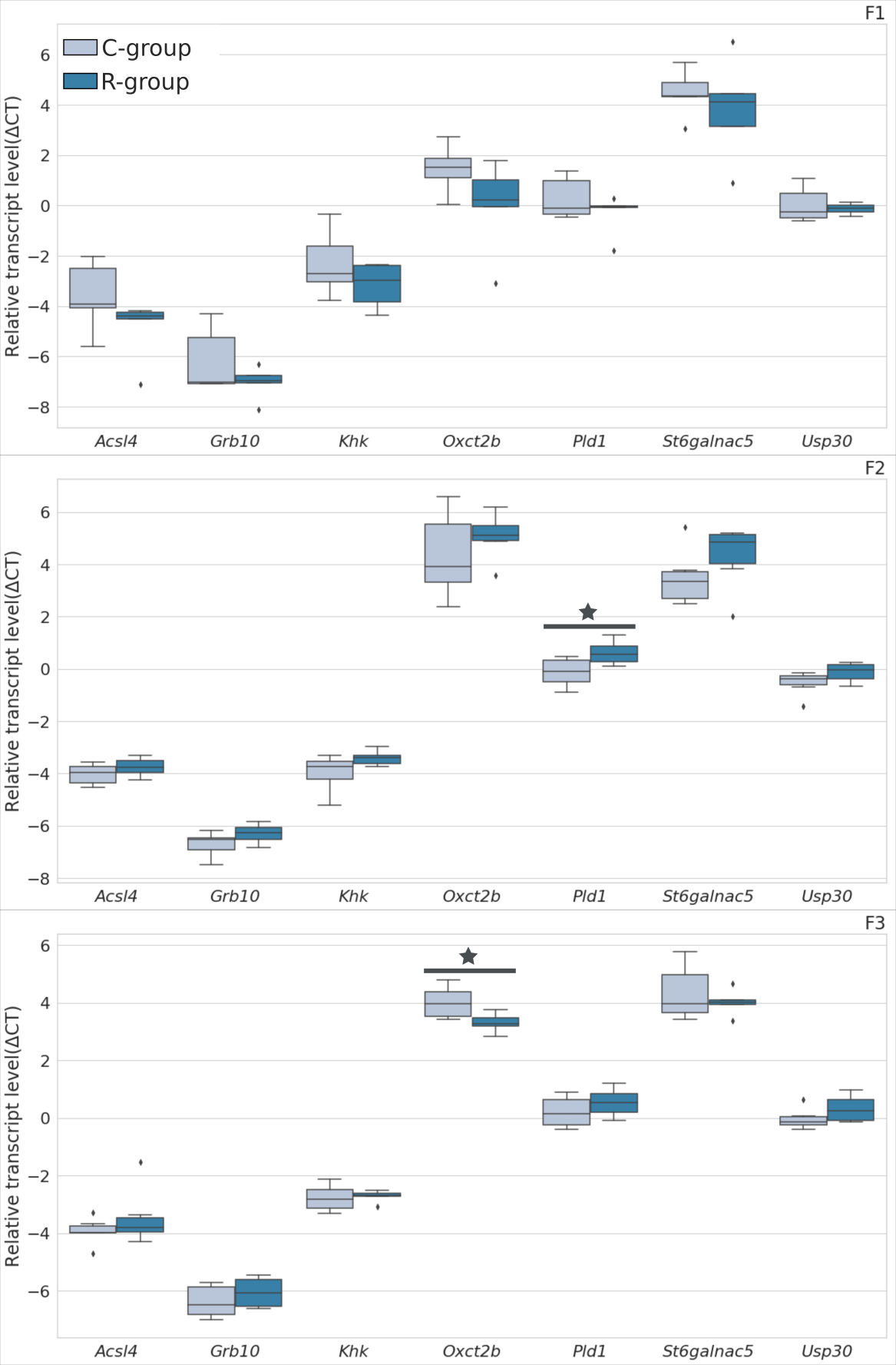

Supplement: S2 File — (DOCX) [file pone.0215471.s002.docx]
